# Impact of Different Types of Nosocomial Infection on the Neurodevelopmental Outcome of Very Low Birth Weight Infants

**DOI:** 10.3390/children8030207

**Published:** 2021-03-09

**Authors:** Karin Pichler, Vito Giordano, Gereon Tropf, Renate Fuiko, Angelika Berger, Judith Rittenschober-Boehm

**Affiliations:** Division of Neonatology, Intensive Care and Neuropediatrics, Department of Pediatrics and Adolescent Medicine, Comprehensive Center for Pediatrics, Medical University of Vienna, Waehringer Gürtel 18-20, 1090 Vienna, Austria; vito.giordano@meduniwien.ac.at (V.G.); n1442255@students.meduniwien.ac.at (G.T.); renate.fuiko@meduniwien.ac.at (R.F.); angelika.berger@meduniwien.ac.at (A.B.); judith.rittenschober-boehm@meduniwien.ac.at (J.R.-B.)

**Keywords:** nosocomial infection, very low birth weight infants, neurodevelopmental outcome

## Abstract

Nosocomial infections (NIs) are important conditions associated with mortality and morbidity in very low birth weight infants (VLBWIs). The aim of this study was to investigate the impact of NIs and the different subtypes on neurodevelopmental outcomes in a cohort of VLBWIs. VLBWIs born with a gestational age between 23 ^0/7^ and 31 ^6/7^ weeks in a level III neonatal center were enrolled. Neonatal morbidities as well as the neurodevelopmental outcome at 2 years of corrected age were analyzed. Six-hundred infants completed the study successfully. Of these, 38% experienced an NI episode. NIs were associated with an increased risk of neonatal complications, such as brain injury, bronchopulmonary dysplasia (BPD) and death, and were a significant risk factor for adverse motor development at 2 years of corrected age in our cohort of VLBWIs. The negative impact of NIs on neurodevelopmental outcomes was particularly associated with necrotizing enterocolitis (NEC), suspected NIs and Gram-positive NIs. This study demonstrated that NIs are a significant risk factor for both morbidity and mortality as well as adverse neurodevelopmental outcomes in VLBWIs.

## 1. Introduction

Improvements in obstetric and neonatal intensive care within the last decade have markedly increased the survival and lowered the border of viability of preterm infants. However, these improvements have not succeeded in reducing the rate of adverse neurodevelopmental outcome of extremely preterm infants [1,2]. 

Nosocomial infections (occurring after 3 postnatal days) are important conditions that are associated with mortality and morbidity as well as poor neurodevelopmental outcomes among infants hospitalized in the neonatal intensive care unit (NICU) [3]. Very low birth weight (VLBW, birth weight <1500 g) infants are at particularly high risk for infections due to multiple factors, including immaturity, especially of the immune system; with decreased function of neutrophils and a low concentration of immunoglobulins; prolonged hospitalization; and the frequent need of invasive procedures, such as endotracheal intubation and intravascular catheterization [3]. Among surviving VLBW infants, 65% experience at least one suspected or culture-proven infection episode during their birth hospitalization, and approximately 35% have a culture-proven nosocomial infection [4]. The mortality following nosocomial infections was reported to be up to 18% [3,4]. The most common pathogens isolated in cases of culture-proven nosocomial infection in VLBW infants are Gram-positive organisms, including coagulase-negative staphylococci, with low virulence and low mortality rates [5]. However, nosocomial infections might also be associated with more virulent pathogens such as *Staphylococcus aureus*, *E. coli* and other Gram-negative aerobes, as well as in rare cases fungi. In some cases, despite the presence of clinical and laboratory signs of infection, no causative pathogen can be identified. This might be attributed to technical issues, primarily the low blood volume available in these small patients. Although a blood volume of 0.5–1.0 mL has been considered sufficient for detecting blood stream infections in neonates and preterm infants, today, researchers presume that the incidence of low-level bacteremia in these patients is more common than previously reported [6]. Infectious episodes without an identified causing pathogen are reported as suspected nosocomial infections. 

The aim of the present study was to investigate the impact of nosocomial infections and its different subtypes on neurodevelopmental outcomes at 2 years of corrected age in a cohort of VLBW infants. Although of multifactorial origin, cases of necrotizing enterocolitis (NEC) were also included in the analysis. This was performed in analogy to former studies on this topic [7,8] and due to the strong association of NEC with infection and inflammation [9]. 

## 2. Materials and Methods

### 2.1. Patients and Data Acquisition

This retrospective analysis included VLBW infants with a gestational age between 23 ^0/7^ and 31 ^6/7^ weeks and birth weight <1500 g, born in our level III neonatal center between January 2009 and June 2016, who were admitted to the NICU. Infants with major congenital malformations/syndromes and congenital metabolic disorders were excluded from the study. 

The basic patient characteristics and clinical data were collected from the medical records of the included infants: the maternal pregnancy history (use of antenatal steroids and mode of delivery), 5-min APGAR score, gestational age at birth, birth weight, sex, single or multiple birth, early onset sepsis (EOS), duration and type of respiratory support, as well as the relevant complications associated with prematurity as defined below. The study was conducted according to the guidelines of the Declaration of Helsinki and approved by the local ethics committee (EK-Nr. 1090/2018).

### 2.2. Definitions

A proven nosocomial infection (pNI) was defined as a positive result of one or more bacterial or fungal isolates obtained from a blood culture in an infant with clinical signs of infection (temperature instability, irritability, apathia, feeding difficulties, prolonged capillary refill, apnea, tachycardia and tachypnea) after day 3 of life treated with antibiotics for 5 or more days or until death. Based on the culture results (Gram-positive pathogen, Gram-negative pathogen or fungus), the infants were classified into three nosocomial infection subgroups. Pathogens that may represent contaminations (coagulase-negative staphylococci) were considered as proven sepsis only if clinical and laboratory signs of infection (at least one of the following: elevated C-reactive protein >2 mg/dL, left shift with an I/T ratio >0.2 or leukopenia <5/nL) were present and if antibiotics/antimycotics were administered for a minimum of 5 days or until death. 

Suspected nosocomial infection (sNI) was defined as an episode with clinical and/or laboratory signs of an infection in the absence of a positive bacterial or fungal culture in an infant who received treatment with antibiotics for a minimum of 5 days or until death.

Cases of NEC Bell stage II or higher [10] with or without bacteremia were included in the study.

Early-onset sepsis (EOS) was defined as culture-proven sepsis within the first 72 h of life.

The uninfected group consisted of infants who had not developed pNI, sNI or NEC as defined above until discharge.

Brain injury was defined as intraventricular hemorrhage grade 3 or higher, according to Deeg et al. [11], and/or cystic periventricular leukomalacia.

Bronchopulmonary dysplasia (BPD) was defined as oxygen dependency at 36 weeks postmenstrual age [12].

### 2.3. Neurodevelopmental Assessment

Neurodevelopmental examination was routinely performed by experienced pediatric developmental neurologists and developmental psychologists at 24 months of corrected age at the neonatal follow-up clinic of the department. Neurodevelopment was assessed using the Bayley Scales of Infant Development II (BSID-II) for those infants born between 1 January 2009 and 30 June 2012 [13]. For those born between 1 July 2012 and 30 June 2016, the BSID-III were used [14]. To compare infants assessed with both scales, the mean of the BSID-III Cognitive Developmental and Language Developmental Index was calculated and compared to the Mental Developmental Index (MDI) of the BSID-II, and the Motor Development Index of the BSID-III was compared to the Psychomotor Development Index (PDI) of the BSID-II. German normative values were used for Bayley-III [15,16]. The mean score for all indices was 100, respectively, with a standard deviation (SD) of 15. Scores < −1 SD relative to the mean were classified as mild delay or impairment, scores < −2 SDs relative to the mean were classified as moderate and scores < −3 SDs relative to the mean as a severe delay or impairment [17]. The indices from BSID-II and Bayley-III were summarized as the Mental Score and Motor Score in this study.

### 2.4. Statistics

Statistical analysis was performed using SPSS^®^ Statistics for Mac version 21.0. (Armonk, NY, USA: IBM Corp.). The quantitative data are presented as the mean ± standard deviation (SD), while the qualitative data are shown as counts and percentages. The differences for continuous variables between the two groups (any type of NI vs. uninfected) were calculated using a *t*-test for independent samples, while differences in the descriptive characteristics for categorial variables were calculated using the chi-square test.

An analysis of variance (ANOVA) was carried out to compare the means between the different NI groups, and a Tukey HSD test was used to calculate the specific differences between these groups. 

A multilinear regression model was performed to investigate the effect of the NI and its subtypes on the neurodevelopmental outcomes when correcting for important patient-specific and/or medical conditions (gender, birth weight, brain injury and BPD). These conditions were chosen due to their high impact on neurodevelopmental outcomes according to the literature [1,18,19,20,21,22,23].

## 3. Results

### 3.1. Study Population

**Figure 1:**
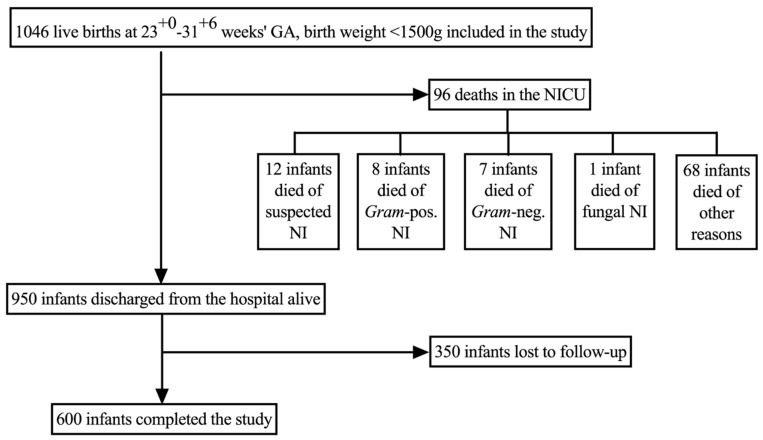
The study cohort.

During the observational period, 1046 infants met the inclusion criteria for the study and were included in the analysis (Figure 1). Ninety-six (9%) infants died during birth hospitalization. No infant included in the study died during the follow-up period. Neurodevelopmental assessment at 2 years of age was not available in 377 (36%) infants, who were considered lost to follow-up. Thus, 600 (57%) infants completed the study successfully (Figure 1). 

The baseline characteristics of the infants who completed the study, those lost to follow-up and those who died during birth hospitalization are provided in Table S1. Briefly, the infants who were lost to follow-up had significantly less NI episodes (*p* = 0.000*), a higher gestational age (*p* = 0.000*) and birth weight (*p* = 0.000*) and less complications associated with prematurity (respiratory support, BPD and brain injury) compared to the infants who completed the study.

Infants who died during birth hospitalization had a higher number of Gram-negative NI (*p* = 0.019*) and NEC (*p* = 0.002*) episodes and a significantly lower gestation age (*p* = 0.000*) and birth weight (*p* = 0.000*), as well as more complications associated with prematurity (respiratory support, BPD and brain injury).

Of the infants who completed the study, 230 (38%) infants experienced an NI episode, while 370 (62%) infants remained uninfected. The baseline characteristics of these infants are given in Table S2. Briefly, the infants who remained uninfected were more frequently delivered via Cesarean section (*p* = 0.001*), had a higher birth weight (*p* = 0.000*) and gestational age (*p* = 0.000*), were more frequently multiples (*p* = 0.023*) and had less complications associates with prematurity (respiratory support, BPD and brain injury). The baseline characteristics of the NI subtypes are given in Table S3.

### 3.2. Influence of Nosocomial Infections on Neurodevelopmental Outcome

The neurodevelopmental outcome assessments were performed at 2 years of corrected age. Both mental score (87 (SD ± 19) versus 80 (SD ± 21); *p* = 0.000*) and motor score (90 (SD ± 15) versus 83 (SD ± 18); *p* = 0.000*) were significantly higher in uninfected infants versus infants with NI in an unadjusted analysis, revealing mild motor and mental impairment in the infants who experienced infectious episodes.

When controlling for neonatal characteristics and morbidities (gender, birth weight, BPD and brain injury), NIs were still significantly associated with an adverse motor (*p* = 0.004*) but not mental outcome (*p* = 0.062) (Table 1).

### 3.3. Influence of Nosocomial Infection Subtypes on the Neurodevelopmental Outcome

In an unadjusted analysis, infants with sNI and infants with episodes of NEC showed a significant, mild impairment in both motor and mental development compared to uninfected infants at 2 years of age. A mild significant impairment in motor development was detected also in infants with a Gram-positive NI (Table 2).

In a regression model, episodes of NEC adversely influenced both the mental and motor development of VLBWIs at 2 years of age (*p* = 0.031 */*p* = 0.025 *; Table 3). 

As the pathophysiological mechanisms of NEC appear to be more complex than in other nosocomial infections, the regression model was also calculated excluding cases of NEC with comparable results (Table S4). 

## 4. Discussion

Nosocomial infections are an important morbidity in very low birth weight infants. In line with other studies [8,21], our data showed that the rates of nosocomial infection increased with decreasing birth weight and gestational age. Moreover, in our study, nosocomial infections were associated with an increased risk of neonatal complications, such as prolonged mechanical ventilation, BPD, brain injury and, most importantly, death. In our cohort of VLBWIs, infants who experienced an episode of nosocomial infection showed a mild motor and mental delay at 2 years of corrected age. In the regression model, nosocomial infections were an independent risk factor for adverse motor outcome. When analyzing the subtypes of nosocomial infections, infants with episodes of suspected nosocomial infection and infants with NEC showed a mild motor and cognitive impairment at 2 years of age, whereas infants with Gram-positive nosocomial infections displayed a mild motor delay at 2 years of age. In the regression model, NEC adversely influenced both the mental and motor development at 2 years of age.

Our results are in line with the current data [23,24] but differ significantly from older studies [8]. 

The large cohort study by Stoll et al. [8] proposed all types of nosocomial infections including NEC as significant risk factors for both mental and motor scores <70 at 2 years of corrected age in extremely low birth weight infants. In a more recent study of the same group, again reporting neurodevelopmental outcome data of extremely low birth weight infants, nosocomial infection was a much weaker and only slightly significant predictor of the combined outcome late death or survival with significant impairment as opposed to the three other common neonatal morbidities BPD, brain injury and severe retinopathy of prematurity (ROP) [18]. In line with our results, nosocomial infection was found to be a risk factor for impaired motor development in a Swiss cohort of extremely preterm infants at 2 years of corrected age but failed to reach significance in a multivariable model, in contrast to BPD, brain injury, ROP and low socioeconomic status [23]. Finally, Zonnenberg et al. could not identify any difference in the neurodevelopmental outcome at 2 years of corrected age between infants with and without culture proven nosocomial infection [24]. The latter study was limited by a relatively small sample size.

Thus, from the published data, it is reasonable to speculate that the influence of nosocomial infection on the neurodevelopmental outcome of very low and extremely low birth weight infants has declined over the last two decades, while other neonatal morbidities, such as BPD, ROP and brain injury, have gained more importance. This might be explained by improvements in neonatal care during these years, favoring less invasive treatment strategies, above all less invasive surfactant administration, the avoidance of mechanical ventilation and a strong focus on family centered care as well as advanced antibiotic stewardship [25]. These innovations have paved the way for a significantly increased survival rate of the smallest and most immature infants. In fact, in our study cohort, where these principles of less-invasive, family-centered care were applied, the mortality rate was only 9%, while all other studies have reported higher mortality rates [8]. Previous studies have suggested that neonatal infections may predispose very preterm infants to develop BPD, brain injury and ROP and, hence, may determine the long-term outcome via these intermediate morbidities [19,26,27,28]. This hypothesis was supported by our data, as those infants who experienced at least one episode of nosocomial infection had significantly higher incidences of BPD, brain injury and severe ROP. The overall incidence of nosocomial infections in our cohort was only 38%, which is lower than in other studies [8]. The lower incidence of nosocomial infections in our cohort compared to other studies might be one explanation for the low impact of nosocomial infections detected on the neurodevelopmental outcome. This might indicate a high awareness for the prevention of nosocomial infections in our team and the success of the preventive strategies that have been implemented in recent years, such as hand hygiene programs and simulation-based trainings of central line placement [29]. 

In our study, infants with suspected nosocomial infection and infants with NEC showed a mild motor and mental delay at corrected 2 years of corrected age. Infants with Gram-positive nosocomial infections displayed a mild motor delay. In the regression model, NEC adversely influenced both the mental and motor development at 2 years of age. Reports on the influence of different types of nosocomial infections on the neurodevelopmental outcomes of preterm infants are sparse and report differing results. One study detected a significant influence of all types of nosocomial infections on neurodevelopmental outcomes [8], while another study found a higher risk of cerebral palsy only in infants with Gram-positive nosocomial infections at 2 years of corrected age [23]. As for NEC, multiple studies have reported a strong association with adverse neurodevelopmental outcomes, particularly cerebral palsy [30,31]. 

The strength of our study is the fact that our study group reflects a representative NICU patient population. These outcomes may change as the children become older, i.e., some may improve, some may remain unchanged and some may worsen [21], and it would be of interest to evaluate the study population again at a later time point, for example, at school age. The major limitation of the study is that it is a retrospective data analysis. However, it includes a relevant and representative number of NICU patients, and the drop-out rates are in line with other studies [8,18,23]. Given that patients seen in our follow-up clinic were in general more severely ill and had higher rates of infection compared to those lost to follow-up (who were more likely to be transferred to other hospitals before discharge), it is unlikely that the high rate of patients lost to follow-up significantly affected the study findings. It could be speculated that a bias might have been introduced by the infants who died before follow-up, especially in those groups, where the mortality rates were high (Gram-negative nosocomial infection and NEC).

In summary, the current study demonstrates that nosocomial infections are a significant risk factor for adverse motor development at 2 years of corrected age in very low birth weight infants as well as a risk factor for neonatal complications, such as brain injury, BPD and death in these infants. The negative impact of nosocomial infections on neurodevelopmental outcomes was particularly associated with NEC, suspected nosocomial infections and Gram-positive nosocomial infections in our study cohort.

## Figures and Tables

**Table 1:** Regression model for the influence of nosocomial infections on neurodevelopmental outcome.

		Regression Coefficient B	Standard Error	Sig.	95%-Confidence Interval		Partial Eta-Square
					Lower	Upper	
Mental Score	Gender (Female)	6.735	1.580	0.000	3.633	9.837	0.030
	Birth weight <1000g	−4.363	1.739	0.012	−7.778	−0.948	0.011
	BPD	−6.917	2.199	0.002	2.598	11.235	0.016
	Brain Injury (Yes)	−9.628	2.969	0.001	3.798	15.459	0.017
	Nosocomial Infection (Yes)	−3.192	1.704	0.062	−0.155	6.539	0.006
Motor Score	Gender (Female)	4.184	1.260	0.001	1.709	6.659	0.018
	Birth weight <1000g	−1.835	1.387	0.186	−4.560	0.889	0.003
	BPD	−5.918	1.754	0.001	2.473	9.363	0.019
	Brain Injury (Yes)	−16.329	2.369	0.000	11.677	20.981	0.074
	Nosocomial Infection (Yes)	−3.962	1.360	0.004	1.291	6.632	0.014

**Table 2:** Mental Developmental Index (MDI) and Psychomotor Development Index (PDI) mean values for specific infection types.

Outcome	Uninfected	Suspected NI	*p*	Gram-pos. NI	*p*	Gram-neg. NI	*p*	NEC	*p*	Fungal NI	*p*
Mental Score, mean (SD)	87 (19)	77 (21)	0.002 *	82 (21)	0.315	86 (18)	1.000	76 (20)	0.027 *	87 (30)	1.000
Motor Score, mean (SD)	90 (15)	82 (20)	0.001 *	84 (17)	0.010 *	91 (16)	1.000	79 (18)	0.009 *	89 (17)	1.000

* Indicates a level of significance of *p* < 0.05.

**Table 3:** Regression model including nosocomial infection subtypes.

		Regression Coefficient B	Standard Error	Sig.	95%-Confidence Interval		Partial Eta-Square
					Lower	Upper	
Mental Score	Gender (Female)	6.977	1.582	0.000	3.870	10.085	0.032
	Birth weight <1000 g	−4.681	1.751	0.008	−8.121	−1.241	0.012
	BPD (Yes)	−6.617	2.205	0.003	2.287	10.948	0.015
	Brain Injury (Yes)	−9.456	2.994	0.002	3.574	15.337	0.017
	Suspected NI (Yes)	−2.974	2.249	0.187	−1.443	7.390	0.003
	*Gram*-positive NI (Yes)	−1.373	1.987	0.490	−2.529	5.275	0.001
	Gram-negative NI (Yes)	−2.729	4.866	0.575	−12.287	6.829	0.001
	NEC (Yes)	−7.432	3.439	0.031	0.678	14.187	0.008
	Fungal NI (Yes)	6.453	9.607	0.502	−25.321	12.414	0.001
Motor Score	Gender (Female)	4.373	1.263	0.001	1.892	6.855	0.020
	Birth weight <1000 g	−2.217	1.398	0.113	−4.964	0.529	0.004
	BPD	−5.775	1.761	0.001	2.318	9.233	0.018
	Brain Injury (Yes)	−16.308	2.391	0.000	11.613	21.003	0.073
	Suspected NI (Yes)	−2.609	1.795	0.147	−0.917	6.135	0.004
	*Gram*-positive NI (Yes)	−3.050	1.586	0.055	−0.065	6.165	0.006
	*Gram*-negative NI (Yes)	−3.121	3.885	0.422	−10.751	4.510	0.001
	NEC (Yes)	−6.175	2.746	0.025	0.782	11.568	0.009
	Funagl NI (Yes)	5.806	7.670	0.449	−20.870	9.258	0.001

## Data Availability

All data requests should be submitted to the corresponding author for consideration. Access to anonymized data may be granted following review.

## Supplementary Materials

The following are available online at https://www.mdpi.com/2227-9067/8/3/207/s1, Table S1. Baseline characteristics of infants, who completed the study vs. infants, who were lost to follow up or died. p-values were calculated towards those infants, who completed the study. Table S2. Baseline characteristics of uninfected infants versus infants with NIs. Table S3 Baseline characteristics of uninfected infants versus infants with suspected, Gram-positive, Gram-negative, fungal NI and NEC. Table S4: Regression Model for Nosocomial Infection Subtypes excluding NEC.
