# Supplementary material for: Impact of Different Types of Nosocomial Infection on the Neurodevelopmental Outcome of Very Low Birth Weight Infants
Source: Children (Basel). 2021 Mar 9;8(3):207. doi: 10.3390/children8030207 (PMC8000764; doi:10.3390/children8030207)
Supplement: Supplementary file 1 [file children-08-00207-s001.pdf]

Supplementary Materials: The following are available online at https://www.mdpi.com/2227-9067/8/3/207/s1.

Table 1. Baseline characteristics of infants, who completed the study vs. infants, who were lost to follow up or died. *p*-values were calculated towards those infants, who completed the study.

Characteristics	Assessed at 2 years of age n=600	Lost to follow up n=350	*p*	Died n=96	*p*
Nosocomial infections (NI)	230/600 (38%)	77/350 (22%)	0.000*	39/96 (40%)	0.463
No NI	370/600 (62%)	273/350 (78%)	0.020*	57/96 (59%)	0.909
Suspected NI	71/600 (12%)	27/350 (8%)	0.015*	13/96 (13%)	0.534
Gram-positive NI	108/600 (18%)	31/350 (12%)	0.000*	7/96 (7%)	0.018*
Gram-negative NI	14/600 (2%)	11/350 (3%)	0.553	6/96 (6%)	0.019*
Fungal NI	4/600 (1%)	0/350 (0%)	0.303	1/96 (1%)	0.383
Antenatal steroids	578/600 (96%)	335/350 (96%)	0.734	89/96 (9%)	0.179
Cesarean delivery	531/600 (89%)	324/350 (93%)	0.164	72/96 (75%)	0.000*
5-min APGAR score, mean (SD)	8 (1)	9 (1)	0.363	8 (1)	0.996
Early onset sepsis (EOS)	11/600 (2%)	11/350 (3%)	0.263	2/96 (2%)	1.000
Birth weight (g)					
Birth weight, mean (SD), g	927 (263)	1091 (249)	0.000*	670 (187)	0.000*
401-500	15/600 (3%)	2/350 (1%)	0.040	15/96 (16%)	0.000*
501-750	169/600 (28%)	38/350 (11%)	0.000*	56/96 (58%)	0.000*
751-1000	193/600 (32%)	81/350 (23%)	0.000*	20/96 (21%)	0.031
1001-1500	223/600 (37%)	229/350 (65%)	0.000*	5/96 (5%)	0.000*
Gestational age (wk)					
Gestational age, mean (SD), wk	27 (2)	28 (2)	0.000*	25 (2)	0.000*
<25	90/600 (15%)	23/350 (7%)	0.000*	49/96 (51%)	0.000*
25-27	272/600 (45%)	91/350 (26%)	0.000*	36/96 (38%)	0.184
28-31	238/600 (40%)	236/350 (67%)	0.000*	11/96 (11%)	0.000*
Male	314/600 (52%)	171/350 (49%)	0.313	55/96 (57%)	0.284
Multiples	217/600 (36%)	164/350 (47%)	0.001*	32/96 (33%)	0.228
Respiratory Support					
Mechanical ventilation	236/600 (39%)	82/350 (23%)	0.000*	89/96 (93%)	0.000*
Mechanical ventilation, mean (SD), days	4 (8)	3 (7)	0.006*	12 (14)	0.000*
Duration of continuous positive airway pressure (CPAP) ventilation, mean (SD), days	22 (17)	13 (14)	0.000*	5 (9)	0.000*
BPD	104/600 (17%)	34/350 (10%)	0.001*	6/96 (6%)	0.028*
Brain Injury	55/600 (9%)	12/350 (3%)	0.000*	50/96 (52%)	0.000*
NEC	33/600 (5%)	8/350 (2%)	0.020*	12/96 (13%)	0.002*
Postnatal steroids	39/600 (7%)	11/350 (3%)	0.000*	10/96 (10%)	0.060

Characteristics	Assessed at 2 years of age n=600	Lost to follow up n=350	*p*	Died n=96	*p*
Nosocomial infections (NI)	197/600 (33%)	77/350 (22%)	0.000*	39/96 (40%)	0.463
No NI	403/600 (67%)	273/350 (78%)	0.020*	57/96 (59%)	0.909
Suspected NI	71/600 (12%)	27/350 (8%)	0.015*	13/96 (13%)	0.534
Gram-positive NI	108/600 (18%)	31/350 (12%)	0.000*	7/96 (7%)	0.018*
Gram-negative NI	14/600 (2%)	11/350 (3%)	0.553	6/96 (6%)	0.019*
Fungal NI	4/600 (1%)	0/350 (0%)	0.303	1/96 (1%)	0.383
Antenatal steroids	578/600 (96%)	335/350 (96%)	0.734	89/96 (9%)	0.179
Cesarean delivery	531/600 (89%)	324/350 (93%)	0.164	72/96 (75%)	0.000*
5-min APGAR score, mean (SD)	8 (1)	9 (1)	0.363	8 (1)	0.996

Early onset sepsis (EOS)	11/600 (2%)	11/350 (3%)	0.263	2/96 (2%)	1.000
Birth weight (g)					
Birth weight, mean (SD), g	927 (263)	1091 (249)	0.000*	670 (187)	0.000*
401-500	15/600 (3%)	2/350 (1%)	0.040	15/96 (16%)	0.000*
501-750	169/600 (28%)	38/350 (11%)	0.000*	56/96 (58%)	0.000*
751-1000	193/600 (32%)	81/350 (23%)	0.000*	20/96 (21%)	0.031
1001-1500	223/600 (37%)	229/350 (65%)	0.000*	5/96 (5%)	0.000*
Gestational age (wk)					
Gestational age, mean (SD), wk	27 (2)	28 (2)	0.000*	25 (2)	0.000*
<25	90/600 (15%)	23/350 (7%)	0.000*	49/96 (51%)	0.000*
25-27	272/600 (45%)	91/350 (26%)	0.000*	36/96 (38%)	0.184
28-31	238/600 (40%)	236/350 (67%)	0.000*	11/96 (11%)	0.000*
Male	314/600 (52%)	171/350 (49%)	0.313	55/96 (57%)	0.284
Multiples	217/600 (36%)	164/350 (47%)	0.001*	32/96 (33%)	0.228
Respiratory Support					
Mechanical ventilation	236/600 (39%)	82/350 (23%)	0.000*	89/96 (93%)	0.000*
Mechanical ventilation, mean (SD), days	4 (8)	3 (7)	0.006*	12 (14)	0.000*
Duration of continuous positive airway pressure (CPAP) ventilation, mean (SD), days	22 (17)	13 (14)	0.000*	5 (9)	0.000*
BPD	104/600 (17%)	34/350 (10%)	0.001*	6/96 (6%)	0.028*
Brain Injury	55/600 (9%)	12/350 (3%)	0.000*	50/96 (52%)	0.000*
NEC	33/600 (5%)	8/350 (2%)	0.020*	12/96 (13%)	0.002*
Postnatal steroids	39/600 (7%)	11/350 (3%)	0.000*	10/96 (10%)	0.060

Table S2. Baseline characteristics of uninfected infants versus infants with NIs.

Characteristics	Uninfected<br>n = 370 (62%)	NI<br>n=230 (38%)	*p*
Antenatal steroids	357/370 (96%)	221/230 (97%)	0.664
Cesarean delivery	344/370 (93%)	187/230 (84%)	0.001*
5-min APGAR score, mean (SD)	8 (1)	8 (1)	0.332
Early-onset sepsis (EOS)	9/370 (2%)	2/230 (1%)	0.219
Birth weight (g)			
Birth weight, mean (SD), g	1004 (247)	802 (237)	0.000*
401-500	4/370 (1%)	11/230 (5%)	0.006*
501-750	67/370 (18%)	102/230 (45%)	0.000*
751-1000	119/370 (32%)	74/230 (31%)	0.929
1001-1500	180/370 (49%)	43/230 (19%)	0.494
Gestational age (wk)			
Gestational age, mean (SD), wk	27 (2)	26 (2)	0.000*
<25	21/370 (6%)	69/230 (30%)	0.000*
25-27	154/370 (42%)	118/230 (51%)	0.023*
28-31	195/370 (52%)	43/230 (19%)	0.000*
Male	188/370 (51%)	126/230 (55%)	0.356
Multiples	147/370 (40%)	70/230 (30%)	0.023*
Respiratory Support			
Mechanical ventilation	93/270 (25%)	143/230 (62%)	0.000*
Mechanical ventilation, mean (SD), days	5 (2)	8 (11)	0.000*
Duration of continuous positive airway pressure (CPAP) ventilation, mean (SD), days	15 (17)	30 (16)	0.000*

BPD	41/370 (11%)	63/230 (27%)	0.000*
Brain Injury	24/370 (6%)	31/230 (14%)	0.001*
Postnatal steroids	8/370 (2%)	31/230 (13%)	0.000*

**Characteristics**	**Uninfected n = 370 (62%)**	**NI n=230 (38%)**	***p***
Antenatal steroids	357/370 (96%)	221/230 (97%)	0.664
Cesarean delivery	344/370 (93%)	187/230 (84%)	0.001*
5-min APGAR score, mean (SD)	8 (1)	8 (1)	0.332
Early-onset sepsis (EOS)	9/370 (2%)	2/230 (1%)	0.219
Birth weight (g)			
Birth weight, mean (SD), g	1004 (247)	802 (237)	0.000*
401-500	4/370 (1%)	11/230 (5%)	0.006*
501-750	67/370 (18%)	102/230 (45%)	0.000*
751-1000	119/370 (32%)	74/230 (31%)	0.929
1001-1500	180/370 (49%)	43/230 (19%)	0.494
Gestational age (wk)			
Gestational age, mean (SD), wk	27 (2)	26 (2)	0.000*
<25	21/370 (6%)	69/230 (30%)	0.000*
25-27	154/370 (42%)	118/230 (51%)	0.023*
28-31	195/370 (52%)	43/230 (19%)	0.000*
Male	188/370 (51%)	126/230 (55%)	0.356
Multiples	147/370 (40%)	70/230 (30%)	0.023*
Respiratory Support			
Mechanical ventilation	93/270 (25%)	143/230 (62%)	0.000*
Mechanical ventilation, mean (SD), days	5 (2)	8 (11)	0.000*
Duration of continuous positive airway pressure (CPAP) ventilation, mean (SD), days	15 (17)	30 (16)	0.000*
BPD	41/370 (11%)	63/230 (27%)	0.000*
Brain Injury	24/370 (6%)	31/230 (14%)	0.001*
Postnatal steroids	8/370 (2%)	31/230 (13%)	0.000*

Table S3. Baseline characteristics of uninfected infants versus infants with suspected, Gram-positive, Gram-negative, fungal NI and NEC.

**Characteristics**	**Uninfected n = 370 (62%)**	**Suspected NI n=71 (12%)**	***p***	***Gram*-pos. NI n=108 (18%)**	***p***	***Gram*-neg. NI n=14 (2%)**	***p***	**NEC n=33 (5%)**	***p***	**Fungal NI n=4 (1%)**	***p***
Antenatal steroids	357/370 (96%)	69/71 (97%)	*0.239*	104/108 (96%)	*0.431*	14/14 (100%)	*1.000*	31/33 (94%)	*1.000*	3/4 (75%)	*0.145*
Cesarean delivery	344/370 (93%)	61/71 (86%)	*0.042**	95/108 (88%)	*0.241*	3/14 (21%)	*0.073*	25/33 (76%)	*0.068*	3/4 (75%)	*0.354*
5-min APGAR score, mean (SD)	8 (1)	8 (1)	*0.986*	8 (1)	*0.997*	8 (1)	*0.998*	8 (1)	*0.976*	8 (1)	*1.000*
Early-onset sepsis	9/370 (2%)	1/71 (1%)	*1.000*	0/108 (0%)	*0.133*	1/14 (7%)	*0.259*	0/33 (0%)	*1.000*	0/4 (0%)	*1.000*
Birth weight (g)											
Birth weight, mean (SD), g	1004 (247)	750 (186)	*0.000**	828 (250)	*0.000**	810 (247)	*0.041*	852 (284)	*0.009**	626 (37)	*0.025*
401-500	4/370 (1%)	5/71 (7%)	*0.005**	5/108 (5%)	*0.092*	1/14 (7%)	*0.337*	0/33 (0%)	*1.000*	0/4 (0%)	
501-750	67/370 (18%)	33/71 (47%)	*0.000**	42/108 (39%)	*0.000**	5/14 (36%)	*0.169*	18/33 (55%)	*0.001**	4/4 (100%)	*0.006**
751-1000	119/370 (32%)	27/71 (38%)	*0.905*	35/108 (33%)	*1.000*	7/14 (50%)	*0.415*	5/33 (15%)	*0.035**	0/4 (0%)	*0.312*
1001-1500	180/370 (49%)	6/71 (8%)	*0.905*	26/108 (24%)	*1.000*	1/14 (7%)	*0.415*	10/33 (30%)	*0.035**	0/4 (0%)	*0.312*

Gestational age (wk)										
Gestational age, mean (SD), wk	27 (2)	26 (2)	*0.000**	26 (2)	*0.000**	26 (2)	*0.016**	26 (2)	*0.018**	25 (2)	*0.107*
<25	21/370 (6%)	26/71 (37%)	*0.000**	29/108 (27%)	*0.000**	4/14 (29%)	*0.076*	8/33 (24%)	*0.133*	2/4 (50%)	*0.109*
25-27	154/370 (42%)	34/71 (48%)	*0.578*	61/108 (57%)	*0.010**	8/14 (57%)	*0.449*	14/33 (43%)	*0.858*	1/4 (25%)	*0.630*
28-31	195/370 (52%)	11/71 (15%)	*0.000**	18/108 (16%)	*0.000**	2/14 (14%)	*0.035**	11/33 (33%)	*0.471*	1/4 (25%)	*1.000*
Male	188/370 (51%)	38/71 (54%)	*0.656*	62/108 (57%)	*0.415*	9/14 (64%)	*0.804*	15/33 (45%)	*0.721*	2/4 (50%)	*1.000*
Multiples	147/370 (40%)	21/71 (30%)	*0.908*	33/108 (30%)	*0.288*	6/14 (43%)	*0.293*	8/33 (24%)	*0.191*	2/4 (50%)	*0.458*
Respiratory Support										
Mechanical ventilation	93/270 (25%)	48/71 (68%)	*0.000**	57/108 (53%)	*0.000**	8/14 (57%)	*0.069*	27/33 (82%)	*0.000**	3/4 (75%)	*0.305*
Mechanical ventilation, mean (SD), d	5 (2)	10 (10)	*0.000**	6 (9)	*0.000**	10 (16)	*0.001**	13 (11)	*0.000**	12 (8)	*0.079*
Duration of CPAP, mean (SD), d	15 (17)	31 (16)	*0.000**	30 (16)	*0.000**	35 (15)	*0.000**	27 (17)	*0.007**	38 (9)	*0.074*
BPD	41/370 (11%)	23/71 (32%)	*0.000**	25/108 (23%)	*0.031**	3/14 (21%)	*0.498*	11/33 (33%)	*0.018**	1/4 (25%)	*0.534*
Brain Injury	24/370 (6%)	20/71 (28%)	*0.000**	13/108 (12%)	*0.193*	2/14 (15%)	*0.371*	4/33 (12%)	*0.325*	1/4 (25%)	*0.284*
Severe ROP	11/370 (3%)	14/71 (20%)	*0.000**	22/108 (20%)	*0.000**	1/14 (7%)	*0.210*	9/33 (27%)	*0.003**	3/4 (75%)	*0.004**
Postnatal steroids	8/370 (2%)	12/71 (17%)	*0.000**	14/108 (13%)	*0.000**	3/14 (21%)	*0.079*	1/33 (3%)	*0.715*	1/4 (25%)	*0.236*

Table S4. Regression Model for Nosocomial Infection Subtypes excluding NEC.

		**Regression coefficient B**	**Standard Error**	**Sig.**	**95%- Confidence interval**		**Partial Eta-Square**
					**Lower**	**Upper**	
Mental Score	Gender (Female)	6,862	1,586	0,000	3,746	9,977	0,031
	Birth Weight <1000g	-4,690	1,757	0,008	-8,141	-1,240	0,012
	BPD (Yes)	-7,085	2,201	0,001	2,761	11,408	0,017
	Brain Injury (Yes)	-9,586	3,003	0,001	3,688	15,485	0,017
	Suspected NI (Yes)	-2,938	2,255	0,193	-1,492	7,368	0,003
	Gram-positive NI (Yes)	-1,374	1,993	0,491	-2,540	5,288	0,001
	Gram-negative NI (Yes)	-2,241	4,876	0,646	-11,818	7,336	0,000
	Funagl NI (Yes)	6,917	9,634	0,473	-25,838	12,005	0,001
Motor Score	Gender (Female)	4,277	1,267	0,001	1,789	6,766	0,019
	Birth Weight <1000g	-2,225	1,403	0,113	-4,981	0,531	0,004
	BPD (Yes)	-6,164	1,758	0,000	2,711	9,617	0,020
	Brain Injury (Yes)	-16,417	2,398	0,000	11,706	21,127	0,074

	Suspected NI (Yes)	-2,579	1,801	0,153	-0,958	6,117	0,003
	Gram-positive NI (Yes)	-3,052	1,592	0,056	-0,074	6,178	0,006
	Gram-negative NI (Yes)	-2,715	3,894	0,486	-10,364	4,934	0,001
	Fungal NI (Yes)	6,191	7,694	0,421	-21,303	8,921	0,001
